# Predicting affective engagement and mental strain from prosodic speech features

**DOI:** 10.3389/fpsyt.2025.1656292

**Published:** 2025-09-19

**Authors:** Vaishnavi Prakash Yache, Laura Moradbakhti, Irene Neuner, Tanja Veselinovic

**Affiliations:** ^1^ Digital Mental Health Lab, Psychiatry, Psychotherapy and Psychosomatic, RWTH Aachen, Aachen, Germany; ^2^ Institute of Neuroscience and Medicine - 4, Forschungszentrum Jülich, Jülich, Germany; ^3^ Department of Psychiatry and Psychotherapy II, LVR-Hospital Cologne, Cologne, Germany

**Keywords:** speech prosody, positive affective engagement, perceived mental strain, machine learning in mental health, prosodic feature extraction, human-computer interaction

## Abstract

**Background:**

Emotional resilience (traditionally defined as the capacity to recover from adversity) and cognitive load (the mental effort for processing information) are critical aspects of mental health functioning. Traditional assessment methods, such as physiological sensors and post-task surveys, often disrupt natural behavior and fail to provide real-time insights. Speech prosody, encompassing pitch, intensity, loudness, and voice activity, offer a non-intrusive alternative for evaluating these psychological constructs. However, the relationship between speech prosody, emotional resilience, and cognitive load remains underexplored, particularly in conversational contexts.

**Objective:**

This study proposes proxy measures for these constructs based on self-reported engagement, enjoyment, boredom, and cognitive effort during dyadic conversation. By leveraging the SEWA (Automatic Sentiment Estimation in the Wild) database, developed through a European research project on emotion recognition, the research seeks to develop machine learning models that correlate speech patterns with subjective self-reports of emotional and cognitive states.

**Methods:**

Prosodic features, such as pitch variation, vocal intensity, and voice activity, were extracted from the SEWA database recordings. These features are then normalized to account for inter-speaker variability and used as predictors in machine learning models. Regression and classification models are employed to correlate speech features with subjective self-reports, which serve as ground truth for Positive Affective Engagement (as a proxy for emotional resilience) and Perceived Mental Strain (as a proxy for cognitive load). Data from English and German speakers are analyzed separately to account for linguistic and cultural differences.

**Outcomes:**

The study establishes a significant relationship between speech prosody and psychological states, demonstrating that Positive Affective Engagement (as a proxy for emotional resilience) and Perceived Mental Strain (as a proxy for cognitive load) can be effectively predicted through prosodic features. Higher emotional resilience is linked to more discernible prosodic patterns in German speech, such as higher loudness and greater voice probability consistency. In contrast, cognitive load prediction remains consistent across English and German datasets.

**Conclusion:**

This research introduces a novel approach for assessing Positive Affective Engagement (as a proxy for emotional resilience) and Perceived Mental Strain (as a proxy for cognitive load) through speech prosody, highlighting the significant impact of language-specific variations. By combining prosodic features with machine learning techniques, the study offers a promising alternative to traditional psychological assessments. The findings emphasize the need for tailored, multilingual models to accurately estimate psychological states, with potential applications in mental health monitoring, cognitive workload analysis, and human-computer interaction. This work lays the foundation for future innovations in speech-based psychological profiling, advancing our understanding of human emotional and cognitive states in diverse linguistic contexts.

## 1 Introduction

Emotional resilience is defined in the psychological literature as the capacity to adapt to and recover from adversity and stress (1). It influences significantly how individuals cope with challenges, regulate emotions, and sustain psychological health in demanding circumstances (2). Emotionally resilient individuals often exhibit behaviors such as sustained engagement, emotional regulation, and cognitive flexibility. These qualities have been studied through self-reports, behavioral observations, and increasingly through physiological or vocal markers (3). In the context of speech, certain vocal characteristics may be indicative of resilient emotional states. (4) demonstrated the possibility of estimating personal resilience from speech and physiological signals. Thereby, the most resilience-relevant features were spectral features, including ones related to the fundamental frequency, auditory spectrum coefficients, Mel Frequency Cepstral Coefficients, spectral slope, spectral flux and spectral harmonicity. Further, (5) were able to demonstrate physiological distress, as opposed to emotional resilience by analyzing 24 vocal characteristics with a machine learning approach. Similarly, one other group successfully used audio-based markers from free speech responses one month post-trauma to accurately classify major depressive disorder (MDD) and post-traumatic stress disorder (PTSD), demonstrating the potential of vocal biomarkers for early mental health diagnosis following traumatic events (6). For instance, consistent vocal prosody, adaptive modulation of pitch and intensity, and sustained voice activity can reflect stable emotional engagement and regulation, which are hallmarks of emotional resilience.

Similarly, cognitive load, traditionally refers to the mental effort required to process and retain information, plays a fundamental role in determining learning efficiency, productivity, and task performance (7). High cognitive load can impair decision-making, hinder performance, and induce stress, while balanced cognitive load demands to promote engagement and effective problem-solving (8). According to Cognitive Load Theory (CLT), load can be intrinsic (task complexity), extraneous (task presentation), or germane (learning effort). High cognitive load is typically associated with slower speech rates, more hesitations, and decreased prosodic variation (9). Cognitive load and resilience also show complex interrelations. Positive association was demonstrated between resilience and both, intrinsic and extraneous cognitive load, (10)]. Also, people with high resilience exhibited better global cognitive status and reduced risk of cognitive impairment, even during stressful or demanding periods (11, 12) On the other side, elevated cognitive load correlates with poor mental health (13).

Despite their significance, assessing emotional resilience and cognitive load remains challenging. Traditional methods such as physiological monitoring (e.g. heart rate variability, galvanic skin response) and post-task self-reports are widely used but often intrusive, expensive, and impractical in real-world settings (14, 15, 16). These techniques interfere with natural behavior and fail to capture real-time fluctuations in emotional and cognitive states. As a result, researchers are increasingly exploring non-intrusive alternatives to measure these psychological constructs in everyday interactions.

Speech is a fundamental mode of human communication and an emerging source of psychological insight. It provides a rich, real-time signal that reflects cognitive and emotional states without disrupting natural interactions (17). Variations in speech prosody, such as pitch, rhythm, intensity, and pause patterns, are directly influenced by underlying psychological conditions (18, 19). For example, individuals under high cognitive load tend to exhibit slower speech rates, increased hesitation, and prolonged pauses due to elevated mental effort (20, 21). Similarly, emotionally resilient individuals may maintain stable pitch variation and consistent speech intensity, reflecting better emotional regulation and adaptability.

Research highlights the growing potential of speech as a non-invasive biomarker for mental health conditions such as depression. The acoustic and temporal characteristics of speech have been shown to correlate strongly with depressive states, enabling automated screening and monitoring in clinical and real world settings (22). For example, (23) demonstrated that the prosodic and spectral features extracted from the speech effectively distinguish depressed individuals from controls, confirming the diagnostic value of the speech. Similarly, (24) and (25) employed speech recognition technology to analyze timing-related features, such as speech rate and pauses, finding significant associations with depression severity. Furthermore, deep learning approaches have recently improved detection accuracy by modeling complex vocal patterns, as evidenced by (26), who developed neural architectures capable of capturing subtle speech characteristics related to depression. These advances support the integration of speech-based assessments into scalable, real-time mental health monitoring platforms.

Previous studies have explored speech-based emotion recognition and cognitive load estimation (21, 27), and prosodic features have been increasingly used to detect neurological and psychological conditions such as depression and schizophrenia (28, 29, 30). Moreover, systematic reviews highlight the growing potential of voice analysis for detecting neurological and mood disorders, emphasizing how emerging artificial intelligence (AI) techniques can uncover objective markers of mental health conditions from speech signals (31, 32). These findings reinforce the promise of non-invasive, speech-based approaches for psychological assessment, particularly for mental health monitoring in both clinical and everyday settings.

Moreover, recent advancements have incorporated semantic information into speech emotion recognition frameworks. By combining semantic and paralinguistic features, models can capture both the content and the expressive nuances of speech, leading to improved performance in emotion detection tasks (33). Multimodal approaches that integrate textual, acoustic, and visual modalities have demonstrated superior accuracy, particularly in capturing complex affective states during natural interactions (34, 35). These frameworks often employ deep learning architectures such as transformers or recurrent networks to model temporal dependencies and contextual cues, enhancing emotion inference over isolated prosodic features alone (36).

Despite these advancements, the interplay between emotional resilience, cognitive load, and prosody remains underexplored, particularly in conversational settings. Understanding this relationship could lead to the development of automated tools for real-time psychological assessment, benefiting mental health diagnostics, educational support systems, and human-computer interaction.

Furthermore, prosodic markers are influenced by linguistic and cultural factors, raising concerns about generalizing speech-based models across different languages. Even though some machine learning models for speech-based detection of neurological and/or psychological disorders already include data from multiple languages (29), the majority of studies focus on one language only (28, 30). This highlights the importance of accounting for language-specific prosodic patterns when developing speech-based detection models, to ensure both reliability and fairness across diverse populations.

These prosodic attributes are then used as predictors in machine learning models to classify or predict subjective self-reports. Techniques such as regression models (for continuous prediction of psychological states) and classification models (for high vs. low resilience or cognitive load) are applied. Additionally, to account for inter-speaker differences, features are normalized, and models are trained separately for English and German speakers, considering linguistic and cultural differences in prosody (28).

This research introduces a novel framework for non-intrusive psychological assessment through voice analysis. The key contributions include:

- Conversational context analysis, unlike traditional speech emotion recognition, this study examines interpersonal dynamics (e.g., agreement, engagement) and their impact on resilience and cognitive load.
- Non-Intrusive psychological profiling, by eliminating the need for physiological sensors or intrusive self-reports, offering a real-time, speech-only approach.
- Cross-language considerations, by developing models that account for linguistic and cultural differences in prosody, providing broader applicability.

Potential applications range from mental health monitoring (e.g., stress and resilience assessment) to real-time cognitive support in education and workplace settings. Furthermore, human-computer interaction systems, such as virtual assistants, could benefit from adaptive responses based on users’ emotional and cognitive states.

By integrating speech prosody with self-reported emotional resilience and cognitive load measures, this research advances our understanding of how voice reflects psychological states. The proposed machine learning framework paves the way for automated, real-time assessment tools that enhance mental health monitoring, learning environments, and human-machine interactions. Through a deeper exploration of prosody’s role in emotional and cognitive processes, this study contributes to the ongoing evolution of voice-based psychological profiling.

## 2 Methodology

### 2.1 Dataset and participants

This study utilizes the SEWA *Sentiment and Emotion in the Wild* dataset, a rich collection of dyadic conversations where participants discuss emotionally evocative advertisements (37). These interactions inherently involve cognitive effort (processing the advertisements) and emotional exchange (responding to a conversation partner), making them an ideal context for examining speech-based indicators of emotional resilience and cognitive load. The SEWA database also provides subjective self-reports, in which participants rate their engagement, emotional arousal, and conversational experience on a scale of -5 to 5. These self-reports serve as ground truth labels for machine learning models.

To facilitate individual-level speech analysis, the conversations were separated into individual speaker recordings, ensuring that each participant’s speech was analyzed independently. For segmentation, the Hugging Face library was used, which provides tools to efficiently process and separate the individual speaker recordings from the dyadic conversations (38). This allowed for independent analysis of each participant’s speech recordings, facilitating accurate emotional and cognitive state assessment.

After segmentation, the dataset consisted of 66 native English-speaking and 64 native German speaking participants, totaling 130 individual recordings. Each participant provided self-reports on their emotional and cognitive states, which served as the ground truth for model training.

The average age of participants in both datasets is relatively similar, with English speakers averaging approximately 34.94 years and German speakers 31.08 years. The gender distribution is balanced in the English dataset (50% male, 50% female) but shows a slight male majority in the German dataset (60.94% male, 39.06% female).

Although the SEWA is publicly available for academic use, access was obtained through formal request to the dataset organizers, and usage adhered to all stated terms and conditions. All recordings are anonymized, and the dataset includes consent from participants for secondary research, ensuring compliance with ethical standards for human data use.

### 2.2 Feature extraction

To assess Positive Affective Engagement (emotional resilience) and Perceived Mental Strain (cognitive load) through speech prosody, multiple acoustic features were extracted from the segmented speech data. Feature extraction was performed using the openSMILE toolkit (17), a widely used, open-source toolkit developed for the extraction of audio features from speech and music signals, which provides robust prosodic feature analysis.

It is particularly renowned for its efficiency in processing large datasets and its applicability in real-time systems. The toolkit provides a comprehensive set of features, including prosodic elements such as pitch, loudness, and voice quality, which are essential for analyzing emotional states in speech (17, 39, 40). In the context of emotion recognition, prosodic features extracted using openSMILE have been instrumental in capturing the nuances of emotional expression. For instance, studies have utilized openSMILE to extract features like fundamental frequency (F0), intensity, and voice quality measures, which are then analyzed to infer emotional states. These features are computed over short time frames and can be aggregated to provide both local and global perspectives on the speaker’s emotional state (27, 41, 42).

The prosodic features computed are listed in **Table 1**.

**Table 1:** Speech feature categories and their descriptions (43, 44, 45).

Category	Features (Abbreviations)	Description
Fundamental Frequency (Pitch)	F0_sma_de_amean_mean, F0_sma_de_skewness_mean	Reflects vocal fold vibration and is linked to emotional engagement.
Intensity & Loudness	Pcm_intensity_sma_amean_mean, pcm_intensity_sma_de_amean_mean, pcm_loudness_sma_amean_mean, Pcm_loudness_sma_de_amean_mean	Measures vocal energy, associated with emotional arousal.
Spectral Features (MFCCs)	mfcc_sma_de[1]_skewness_mean, mfcc_sma_de[2]_skewness_mean	Mel-frequency cepstral coefficients (MFCCs) capture timbre and vocal tone, which vary with cognitive and emotional states.
Temporal & Voice Activity	Pcm_zcr_sma_amean_mean, voiceProb_sma_amean_mean	Zero-crossing rate (ZCR) measures the frequency of signal sign changes, reflecting rhythm and articulation rate, while voice probability (VoiceProb) estimates the likelihood of speech being voiced rather than silent or unvoiced, offering insights into vocal activity and mental effort.

We selected a focused set of acoustic features (e.g., F0, intensity, MFCCs, temporal and voice activity) based on prior literature indicating their relevance to emotional and cognitive states (9, 46). This approach balances interpretability and reduces the risk of overfitting, particularly when working with moderate-sized datasets.

All extracted features were normalized to account for inter-speaker variability, ensuring consistency across different voices and linguistic backgrounds.

### 2.3 Annotation and ground truth labels

The SEWA dataset includes subjective self-reports from participants, which were used as proxies, ground truth labels for model training and evaluation of psychological states.

Participants rated their emotional and cognitive experiences on a scale of -5 to 5 across dimensions.

While these do not match formal definitions in clinical psychology, we operationalized, these self-reports to create two target variables,:

- Positive Affective Engagement (as a proxy for Emotional Resilience): As the aggregation of participant’s affective positivity and engagement during the interaction (engagement + enjoyment + positive feelings), reflecting affective adaptability within the conversational context. While not equivalent to clinical definitions of emotional resilience — which require adaptation to adversity — this score served as a proxy for momentary emotional adaptability in a socially interactive context.
- Perceived Mental Strain (as a proxy for Cognitive Load): This approach reflects the extent of mental strain or discomfort reported during the conversation (negative feelings − engagement − enjoyment). However, we acknowledge that this proxy does not align directly with traditional definitions of cognitive load, which emphasize task complexity and working memory demands (47). Thus, our cognitive load metric should be interpreted as a subjective impression of effortful or aversive cognitive experience.

The processed labels were then integrated into the dataset alongside the extracted prosodic features, forming the input for machine learning models. By leveraging both binary and multi-class categorization, the study ensured flexibility in predictive modeling, allowing for both high-level classification and nuanced regression analysis. This approach not only strengthened the interpretability of the models but also facilitated a more comprehensive understanding of how speech prosody correlates with cognitive and emotional resilience in real-world conversations.

### 2.4 Machine learning model development

To assess Positive Affective Engagement (emotional resilience) and Perceived Mental Strain (cognitive load) from speech prosody, we implemented a machine learning pipeline using Support Vector Machines (SVM) for classification and linear regression for continuous score prediction. This approach aligns with established methodologies in affective computing and speech-based psychological assessment, where SVMs have been widely used due to their robustness in high-dimensional feature spaces and ability to handle non-linear patterns (27). The methodology consists of feature preprocessing, dimensionality reduction, classification, and evaluation.

The models were developed with the following workflow:

1. Feature Preprocessing and Dimensionality Reduction: Speech prosodic features extracted from the SEWA dataset were standardized using the StandardScaler to mitigate inter-speaker variability. Principal Component Analysis (PCA) was then applied to reduce dimensionality while preserving the most informative variance in the data. The top three principal components were retained as feature representations for subsequent modeling.
2. Data Merging and Categorization: Self-reported emotional Positive Affective Engagement (emotional resilience) and Perceived Mental Strain (cognitive load) scores were used as ground truth. The self-reports were divided into high and low categories based on quantile thresholds. Scores above the 66th percentile were categorized as high, while those below the 66th percentile were labeled as low. The categorized data was merged with the PCA-transformed features, creating a structured dataset for classification and regression analysis. This approach was chosen to preserve a larger portion of the dataset for analysis while still creating distinguishable classes. Compared to stricter splits, which reduce the dataset to 66% of its original size, the 66th percentile method allows better data utilization and model generalization. Additionally, this strategy employs a moderate threshold to strike a balance between ensuring adequate class separation and retaining sufficient data for reliable model training and evaluation (48, 49).
3. Classification and Regression Models: For classification tasks, we trained SVM models to predict binary labels for emotional resilience and cognitive load. The dataset was split into training (80%) and testing (20%) sets, ensuring stratification for balanced class representation. Feature normalization was reapplied to maintain consistency across training and testing data. Additionally, linear regression models were trained to predict continuous self-report scores, allowing for a more granular assessment of psychological attributes.
4. Model Evaluation: Performance metrics, including accuracy, precision, recall, and F1-score, were computed for classification models, while regression performance was assessed using standard error metrics. The models were trained and validated separately for English and German datasets to account for linguistic variations in prosody. To account for variability in model performance due to limited sample size, we applied bootstrapping with 1,000 iterations for both regression and classification tasks. In each iteration, the data were resampled with replacement, followed by model training and evaluation on a stratified test split. This allowed us to compute 95% confidence intervals for key metrics (e.g., accuracy, precision, F1-score), providing a more robust estimate of generalization performance than a single train-test split. Bootstrapping was particularly valuable for assessing the stability and reliability of model predictions across language groups. The results demonstrated the feasibility of using speech features to infer emotional resilience and cognitive load, highlighting the potential of non-intrusive psychological assessment through voice analysis.

### 2.5 Cross-language analysis

Given the linguistic and cultural differences in prosody, separate models were trained and validated for English and German speakers. This ensured that variations in speech patterns due to language differences did not bias the results. The comparative analysis between the two language groups provided insights into the universality of prosodic indicators for psychological assessment. By isolating language groups, the models more accurately capture prosodic markers relevant within each linguistic context, improving prediction robustness.

### 2.6 Ethical considerations

The study adhered to ethical guidelines for working with human speech data. The SEWA data set was used in accordance with its licensing agreements, ensuring participant anonymity and privacy. Since the study involves secondary data analysis, no direct interaction with participants was required. The dataset includes participant consent for secondary research and is fully anonymized. As no identifiable information was used and no new data were collected, additional ethics approval was not required according to standard practices for secondary anonymized data analysis.

## 3 Results

The primary aim of this study was to investigate the relationship between speech prosody and psychological constructs like Positive Affective Engagement and Perceived Mental Strain, using a combination of subjective self-reports and prosodic features from the SEWA database. The results presented in this section provide empirical evidence of how speech characteristics such as pitch, intensity, loudness, spectral features and voice activity can serve as indicators of an individual’s emotional resilience and cognitive load.

### 3.1 Descriptive statistics of participant demographics

The study utilizes a subset of the SEWA dataset, which includes dyadic conversations in English and German. The dataset consists of 66 participants in the English subset and 64 participants in the German subset, as summarized in **Table 2**.

**Table 2:** Comparison of English and German datasets.

Category	English dataset	German dataset
Number of Participants	66	64
Average Age	34.94	31.08
Gender Distribution	50% male, 50% female	60.94% male, 39.06% female

**Figure 1:**
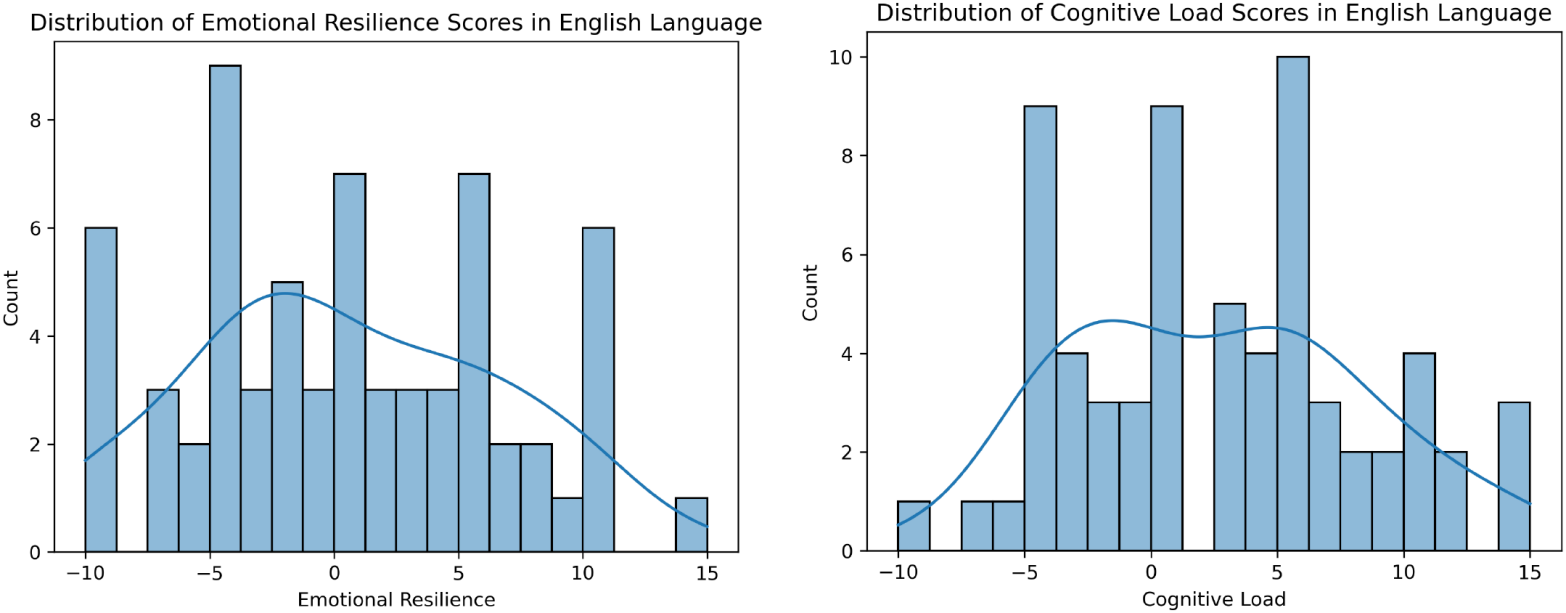
Distribution of ground truth scores for Positive Affective Engagement (left) and Perceived Mental Strain (right) in the English dataset. Histograms show participant self-reports, with kernel density estimates overlaid to illustrate score distributions.

**Figure 2:**
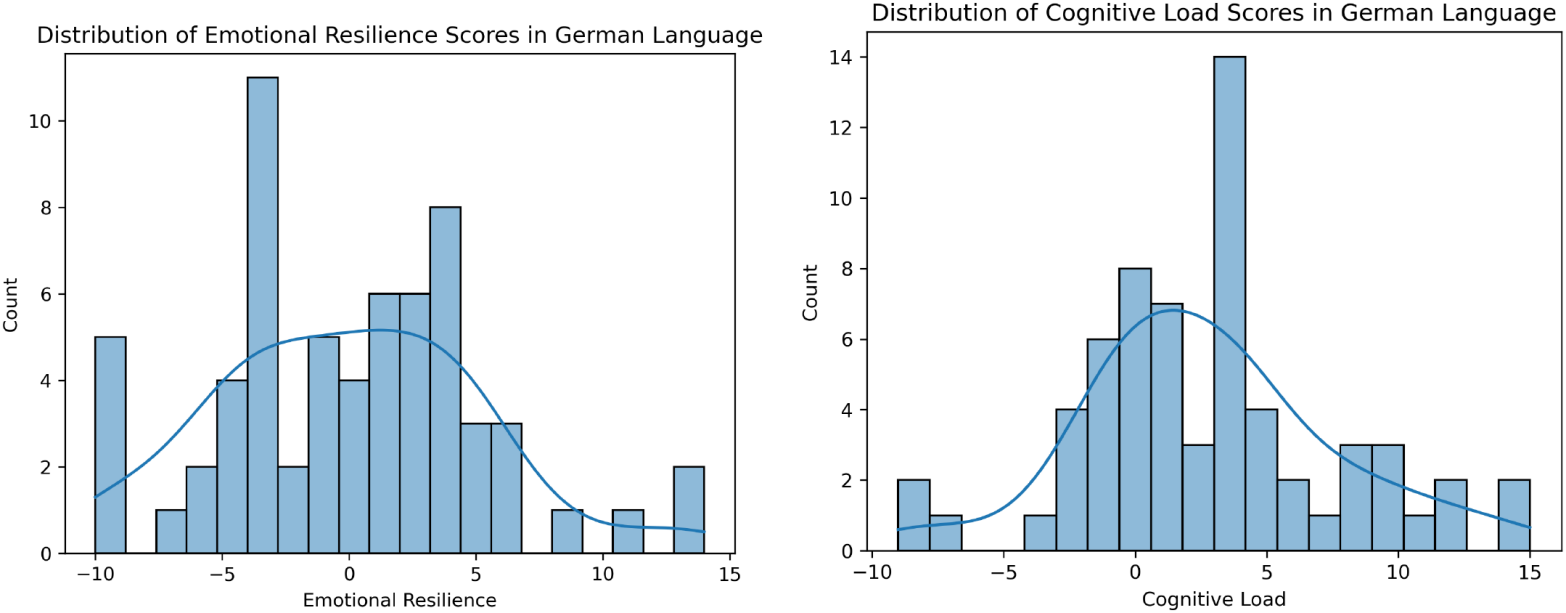
Distribution of ground truth scores for Positive Affective Engagement (left) and Perceived Mental Strain (right) in the German dataset. Histograms show participant self-reports, with kernel density estimates overlaid to illustrate score distributions.

The histograms in **Figures 1**, **2** illustrate the distributions of Positive Affective Engagement (emotional resilience) and Perceived Mental Strain (cognitive load) scores for English and Germanspeaking participants, respectively. These distributions provide insights into how individuals from different linguistic backgrounds perceive and report their psychological states.

Both groups display multimodal distributions, indicating diverse experiences of Positive Affective Engagement (emotional resilience). English speakers tend to report slightly higher resilience on average, with a more balanced spread between positive and negative values. German speakers show a concentration of scores in the negative range, potentially indicating a more critical self-assessment or cultural differences in reporting resilience. German participants report slightly higher Perceived Mental Strain (cognitive load) on average, with fewer instances of extremely low values. English participants show a more evenly distributed pattern, including both high and low cognitive load responses. The stronger peak around 5 in the German dataset suggests a potential cultural or linguistic difference in task perception or self-reporting tendencies. These differences might stem from cultural factors, language-specific prosodic variations, or differing interpretations of the rating scales.

This analysis provides a foundational understanding of how participants self-assess emotional and cognitive states, setting the stage for further statistical comparisons and machine learning modeling.

### 3.2 Feature extraction and visualization

To understand the relationship between speech prosody and psychological states, various acoustic features were extracted (using OpenSMILE) and analyzed for both English and German speakers. **Table 3** summarizes the mean and standard deviation of key prosodic features, categorized into fundamental frequency, intensity & loudness, spectral features, and temporal & voice activity parameters.

**Table 3:** Mean and standard deviation of prosodic features.

Feature category	Feature name	English	German
Fundamental frequency			
F0 (Hz)	F0_sma_de_amean_mean	-0.1895 ± 0.1830	0.0129 ± 0.0076
F0_Skew	F0_sma_de_skewness_mean	0.0102 ± 0.0038	-0.0079 ± 0.0084
Intensity, loudness			
Intensity (mdB)	pcm_intensity_sma_amean_mean	67.8 ± 195.5	73.6 ± 170.9
Intensity_Δ (mdB)	pcm_intensity_sma_de_amean_mean	167.4 ± 262	55.6 ± 170.2
Loudness (dB)	pcm_loudness_sma_amean_mean	0.8432 ± 0.0200	0.9238 ± 0.0596
Loudness _Δ (mdB)	pcm_loudness_sma_de_amean_mean	19.54 ± 115.24	507.3 ± 1571.9
Spectral features			
MFCC1_Skew	mfcc_sma_de[1]_skewness_mean	0.1848 ± 0.0380	-0.0564 ± 0.0475
MFCC2_Skew	mfcc_sma_de[2]_skewness_mean	-0.3539 ± 0.0748	-0.2348 ± 0.0476
Temporal, voice activity			
ZCR	pcm_zcr_sma_amean_mean	0.0728 ± 0.0044	0.0583 ± 0.0107
VoiceProb	voiceProb_sma_amean_mean	0.5653 ± 0.0136	0.6329 ± 0.0090

This table compares prosodic feature measurements (mean ± standard deviation) between English and German speech samples. Abbreviations: F0, Fundamental Frequency; F0 Skew, Skewness of Fundamental Frequency; Intensity, Root Mean Square (RMS) Intensity; Intensity _Δ_, Derivative of Intensity; Loudness, Perceived Loudness in Decibels (dB); Loudness _Δ_, Derivative of Loudness; MFCC1 Skew, Skewness of the 1st Mel-Frequency Cepstral Coefficient; MFCC2 Skew, Skewness of the 2nd Mel-Frequency Cepstral Coefficient; ZCR, Zero-Crossing Rate; VoiceProb, Probability of Voice Activity. Intensity values are based on openSMILE feature extraction. The unit “mdB” is used for readability, values should be interpreted as relative intensity.

1. Fundamental Frequency (Pitch Variability).
  - The F0 mean derivative (F0 sma de amean mean) is slightly negative for English speakers (0.1895 Hz) but positive for German speakers (0.0129 Hz), suggesting that English speakers exhibit greater pitch fluctuations, which may indicate more dynamic intonation. (This feature represents how rapidly pitch changes on average; larger absolute values suggest greater pitch movement over time.).
  - The F0 skewness (F0 sma de skewness mean) is positive for English speakers (0.0102) but negative for German speakers (-0.0079), implying that English speech may be more varied in tone, while German speech has a more balanced pitch distribution. (Skewness reflects asymmetry in the pitch distribution—positive values indicate a longer tail on the right, suggesting more high-pitched variations.).
2. Intensity & Loudness.
  - The mean intensity (pcm intensity sma amean mean) is higher in German speakers (73.6 mdB) than in English speakers (67.8 mdB), indicating that German speech tends to be louder overall. (Intensity corresponds to the energy or perceived volume of the signal, measured in decibels.).
  - The intensity derivative (pcm intensity sma de amean mean) shows a larger fluctuation for English speakers (167.4 mdB) compared to German speakers (55.6 mdB), suggesting that English conversations exhibit more dynamic loudness variations. (The derivative indicates how quickly loudness changes over time—larger values reflect greater loudness modulation.).
3. Spectral Features (MFCC Analysis).
  - The skewness of the first Mel-Frequency Cepstral Coefficient (mfcc sma de[1] skewness mean) is positive for English speakers (0.1848) but negative for German speakers (-0.0564), indicating different spectral energy distributions. (MFCC1 captures coarse spectral shape; its skewness reveals whether the energy distribution leans toward higher or lower frequencies.) (50).
  - The second MFCC skewness (mfcc sma de[2] skewness mean) is lower in German speakers (-0.2348) compared to English speakers (-0.3539). (MFCC2 reflects finer spectral details; negative skewness indicates more concentration of energy in lower coefficients, possibly linked to vowel or consonant articulation styles.) (50).
4. Temporal & Voice Activity Features.
  - Zero-crossing rate (pcm zcr sma amean mean) is higher in English speech (0.0728) than in German speech (0.0583), indicating a more frequent transition between voiced and unvoiced speech sounds in English. (The zero-crossing rate reflects how often the audio waveform crosses the zero amplitude line, i.e., switches from positive to negative or vice versa—and is typically higher in unvoiced or noisy segments.).
  - Voice probability (voiceProb sma amean mean) is higher in German speakers (0.6329) than in English speakers (0.5653), suggesting that German speakers maintain continuous speech more consistently than English speakers. (Voice probability estimates the likelihood that speech (vs. silence or noise) is present at each moment in the signal.).

**Figure 3:**
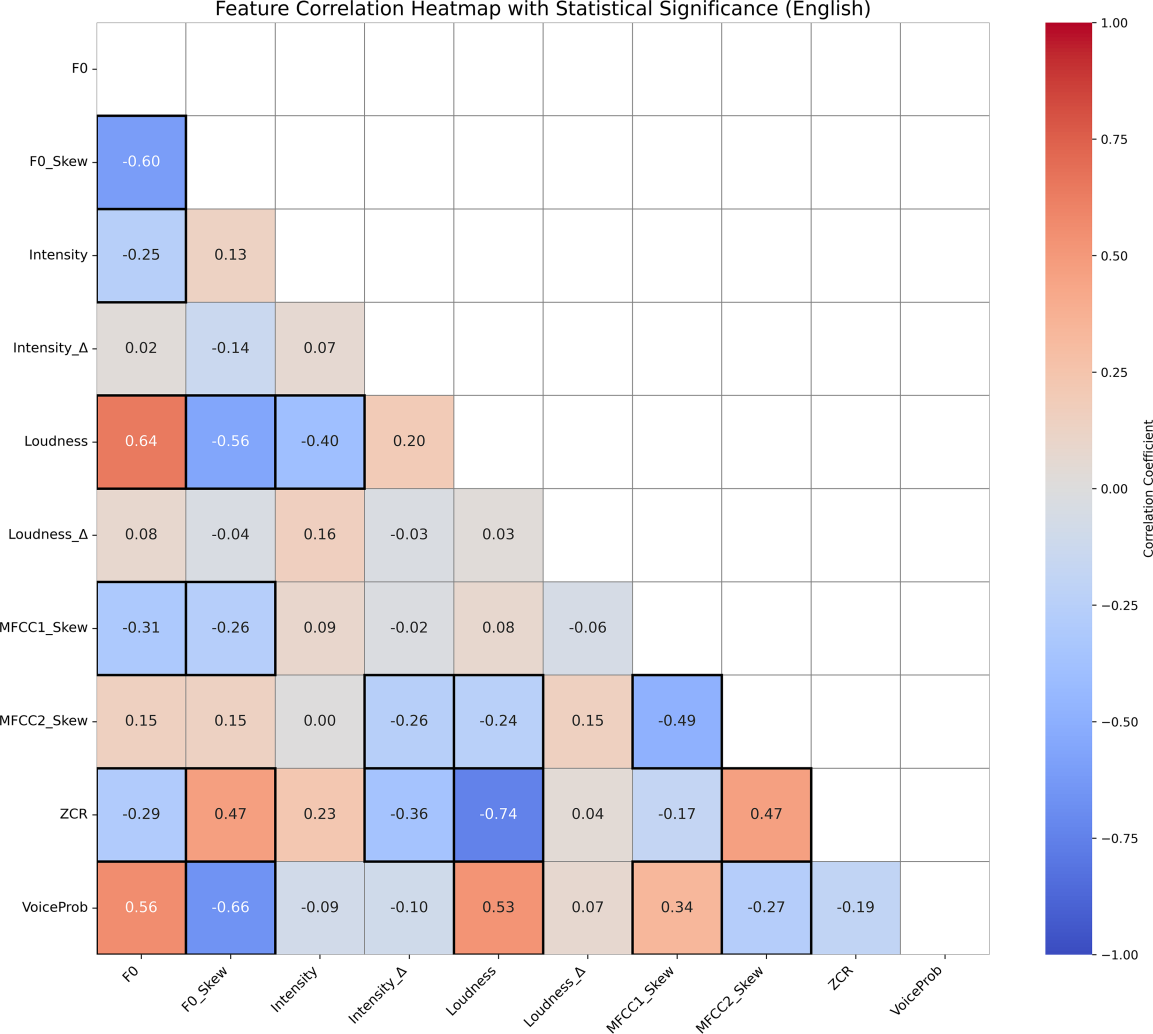
Correlation heatmap of prosodic features in English speech, with Pearson coefficients (–1 to 1) shown by color and significant correlations (*p <* 0.05) marked by boxes, highlighting strong associations.

**Figure 4:**
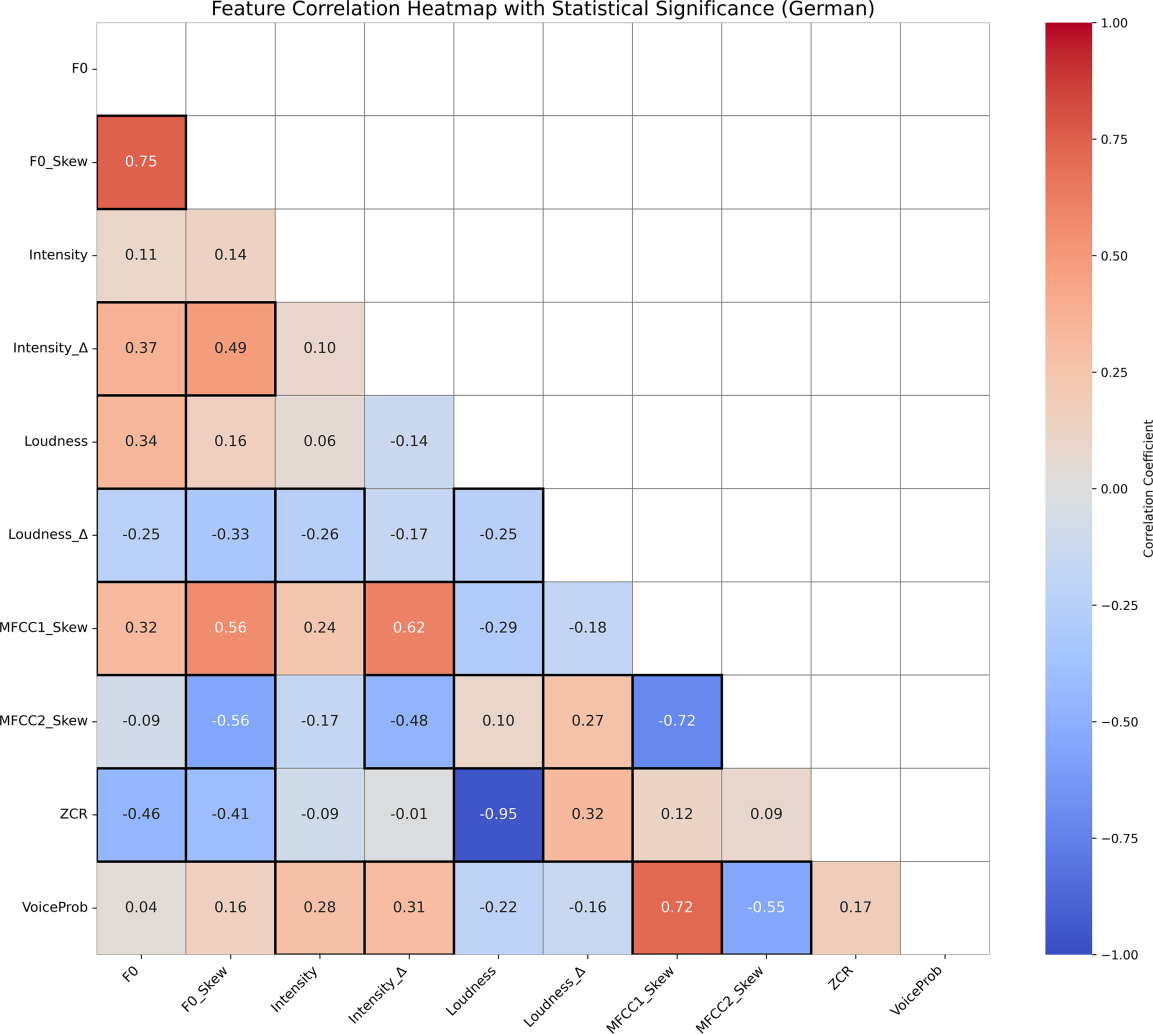
Correlation heatmap of prosodic features in German speech, with Pearson coefficients (–1 to 1) shown by color and significant correlations (*p <* 0.05) marked by boxes, highlighting strong associations.

**Figures 3**, **4** display the Pearson correlation heatmaps of extracted prosodic features in the English and German datasets, respectively. The color scale represents correlation coefficients (r) ranging from -1 (strong negative correlation) to 1 (strong positive correlation), with 0 indicating no correlation. Statistically significant correlations (*p <* 0.05) are marked with highlighted boxes, marking feature relationships that are unlikely to have occurred by chance.

Overall, the German dataset exhibits stronger and more numerous significant correlations between features, suggesting a more tightly integrated prosodic structure. Notably, Loudness and ZCR show a remarkably strong negative correlation in German (*r* =−0.95, *p <* 0.001), but not in English, indicating substantial inter-feature dependency in German speech. This strong inverse relationship can be explained by their distinct acoustic roles: Loudness reflects the perceived intensity of a sound, while ZCR captures how noisy or erratic the signal is. For instance, vowel sounds are often loud yet smooth, resulting in low ZCR, whereas soft background noise may be quiet but chaotic, yielding high ZCR. As such, the two features don’t necessarily increase together—particularly in German, where clearer, vowel-rich articulation may produce speech that is simultaneously louder and less noisy, reinforcing this negative correlation.

Additional differences are observed in the relationships between spectral features (MFCC1 Skew, MFCC2 Skew) and pitch-based metrics (F0 Skew, Intensity). For example, MFCC1 Skew is positively correlated with F0 Skew (*r* = 0.56, *p <* 0.05) and Intensity Δ (*r* = 0.62, *p <* 0.05) in German but shows weaker and inconsistent correlations in English. These differences underscore language-specific acoustic patterns that likely influence model performance.

Moreover, VoiceProb—representing voice activity—exhibited moderate correlations with several features in English (e.g., F0, *r* = 0.56, *p <* 0.05; Loudness *r* = 0.53, *p <* 0.05), while showing weaker and more selective correlations in German, particularly with spectral skew measures (for example, MFCC1 Skew *r* = 0.72, *p <* 0.05; MFCC2 Skew(*r* =−0.55, *p <* 0.05). This variation suggests that voice activity may be cued differently across languages, influencing how features are weighted during model training and learning.

Principal Component Analysis revealed that the top three components captured a substantial portion of variability in prosodic features: 65.74% for English (PC1: 33.93%, PC2: 18.16%, PC3: 13.65%) and 72.93% for German (PC1: 35.67%, PC2: 25.57%, PC3: 11.69%). The dominant features contributing to PC1 in the English dataset were loudness (0.483), pitch skewness (0.440), zero-crossing rate (0.403), and voice probability (0.392). In the German dataset, PC1 was most influenced by spectral skewness (MFCC1: 0.444, MFCC2: 0.405), pitch skewness (0.435), and intensity dynamics (0.368). These results suggest that PCA retained components with clear links to voice expressivity and speech rhythm, preserving psychological interpretability even after dimensionality reduction.

### 3.3 Assessment of positive affective engagement and perceived mental strain from speech (machine learning)

This section presents the predictive performance of Linear Regression for regression tasks and Support Vector Machine (SVM) for classification. The results highlight differences between the English and German datasets in terms of predicting Emotional Resilience and Cognitive Load.

**Table 4** displays the Mean Squared Error (MSE) for Positive Affective Engagement and Perceived Mental Strain across the English and German datasets, using both standard regression evaluation and bootstrapped confidence intervals. In the standard evaluation, the German dataset yields a lower MSE for Positive Affective Engagement (27.146) compared to the English dataset (35.583), suggesting a better model fit for German speakers. For Perceived Mental Strain, the MSE is slightly lower for the English dataset (26.01) than for German (28.52), indicating relatively similar predictive performance across languages.

**Table 4:** Linear regression results for Positive Affective Engagement and Perceived Mental Strain.

Model	Metric	English dataset	German dataset
Positive Affective Engagement	Mean Squared Error (MSE)	35.583	27.146
	Bootstrapped MSE (95% CI)	41.76 [17.63, 92.90]	27.39 [10.54, 51.68]
Perceived Mental Strain	Mean Squared Error (MSE)	26.01	28.52
	Bootstrapped MSE (95% CI)	36.75 [15.16, 74.23]	23.74 [6.85, 47.11]

The bootstrapped results further illustrate the uncertainty around these estimates. For Positive Affective Engagement, the German model achieves a bootstrapped MSE of 27.39 with a narrower 95% confidence interval [10.54, 51.68], whereas the English model shows a higher bootstrapped MSE of 41.76 and a wider confidence interval [17.63, 92.90], reflecting greater variability. Similarly, for Perceived Mental Strain, the bootstrapped MSE is lower in the German dataset (23.74; 95% CI: [6.85, 47.11]) compared to the English dataset (36.75; 95% CI: [15.16, 74.23]). These findings reinforce that Positive Affective Engagement is predicted more reliably in the German dataset, while Perceived Mental Strain shows greater uncertainty in the English dataset. The tighter confidence intervals in the German data may indicate more consistent prosodic cues or less variation in self-reporting among German speakers.

**Tables 5**, **6** report the classification performance for Positive Affective Engagement and Perceived Mental Strain across English and German datasets. Both standard evaluation metrics (based on a single train-test split) and bootstrapped results (with 95% confidence intervals) are presented for a more robust and reliable assessment of model generalization.

**Table 5:** Classification performance for Positive Affective Engagement in English and German datasets (standard and bootstrapped).

Metric	English		German	
	Standard	Bootstrapped	Standard	Bootstrapped
Accuracy	0.429	0.622 [0.357–0.857]	0.615	0.621 [0.308–0.846]
Macro Precision	0.378	0.611 [0.275–0.909]	0.608	0.623 [0.292–0.900]
Macro Recall	0.377	0.597 [0.325–0.854]	0.613	0.616 [0.333–0.875]
Macro F1-score	0.378	0.579 [0.300–0.845]	0.607	0.590 [0.291–0.845]
Confusion Matrix	[5, 4][4, 1]	–	[5, 3][2, 3]	–

**Table 6:** Classification performance for Perceived Mental Strain in English and German datasets (standard and bootstrapped).

Metric	English		German	
	Standard	Bootstrapped	Standard	Bootstrapped
Accuracy	0.571	0.585 [0.286–0.857]	0.538	0.609 [0.308–0.846]
Macro Precision	0.542	0.581 [0.250–0.875]	0.548	0.607 [0.278–0.889]
Macro Recall	0.521	0.570 [0.312–0.833]	0.550	0.592 [0.325–0.845]
Macro F1-score	0.475	0.542 [0.271–0.825]	0.535	0.569 [0.291–0.838]
Confusion Matrix	[7, 1][5, 1]	–	[4, 4][2, 3]	–

For Positive Affective Engagement Classification, the German dataset consistently outperforms the English dataset across all metrics, with notably higher Accuracy (0.615 vs. 0.428), Macro Precision (0.608 vs. 0.378), and Macro F1-score (0.607 vs. 0.378). These results suggest that the classifier was more effective in distinguishing between high and low positive affective engagement in the German speech data, possibly due to language-dependent acoustic patterns or cultural response tendencies. Bootstrapped results confirm this trend but reveal greater uncertainty, particularly in the English dataset. For example, the bootstrapped Accuracy for English is 0.622 with a wide confidence interval [0.357–0.857], compared to 0.621 [0.308–0.846] for German. Despite similar means, the English model exhibits a broader range, indicating less stable generalization. German bootstrapped Macro F1-score (0.590 [0.291–0.845]) also edges out the English value (0.579 [0.300–0.845]), reinforcing the model’s slightly better reliability on German speech.

For Perceived Mental Strain, classification performance is more balanced between languages. The English dataset achieves marginally higher standard Accuracy (0.571 vs. 0.538), while the German dataset outperforms in Macro Recall (0.55 vs. 0.521) and Macro F1-score (0.535 vs. 0.475). Bootstrapped results echo this pattern: English Accuracy averages 0.585 [0.286–0.857], whereas German reaches 0.609 [0.308–0.846]. Notably, German bootstrapped precision (0.607 [0.278–0.889]) and F1-score (0.569 [0.291–0.838]) again exceed those of English, suggesting better balance between sensitivity and specificity.

In summary, the results indicate that language plays a crucial role in speech-based psychological assessments, with machine learning models demonstrating different levels of performance on English and German datasets. Overall, the inclusion of bootstrapping highlights the importance of evaluating model stability under data resampling, especially with modest sample sizes. Positive Affective Engagement classification remains clearly stronger for German, supported by both higher point estimates and tighter confidence intervals. Perceived Mental Strain classification is more variable but shows comparable performance across languages. These results emphasize that language-specific acoustic and reporting factors influence both raw model accuracy and its statistical reliability, underscoring the need for culturally aware and multilingual modeling in speech-based psychological assessment.

## 4 Discussion

In this study, we operationalized Positive Affective Engagement (emotional resilience) and Perceived Mental Strain (cognitive load) using self-report data from the SEWA dataset. Our measure of emotional resilience was derived from participants’ self-reported engagement, enjoyment, and positive affect in response to emotionally evocative advertisements. This operationalization does not aim to capture trait-level resilience, as defined in psychological literature, but instead reflects momentary affective engagement within conversational settings. The Perceived Mental Strain is calculated as a composite of self-reported cognitive effort, boredom, and enjoyment. These refinements improve theoretical coherence while acknowledging the limitations of using self-reports as proxies for complex psychological constructs.

The findings of this study highlight the role of speech prosody in psychological assessments, emphasizing how language-specific variations impact predictive modeling of Positive Affective Engagement and Perceived Mental Strain. The observed differences between English and German datasets suggest that prosodic features contribute uniquely to psychological state estimation, necessitating careful consideration in multilingual applications.

The results demonstrate that Positive Affective Engagement is more accurately predicted in German speech using linear regression, whereas Perceived Mental Strain prediction remains consistent across languages. One possible explanation for this difference lies in the prosodic variations between English and German speakers. German speech exhibited higher loudness and greater consistency in voice probability, potentially making Positive Affective Engagement more discernible through acoustic features. The stronger correlations among prosodic features in German further support this notion, indicating a more tightly connected acoustic profile that facilitates model learning. These observations align with findings by (51), who showed that while German and English share similar prosodic structures, native listeners perceive and weight prosodic cues differently, German listeners being more sensitive to pitch rises and English listeners more to pitch falls. This difference in perceptual sensitivity suggests that prosodic cues are encoded and utilized distinctly across languages, which may contribute to the varying predictive performance seen in our models.

The classification results reinforce this observation, showing that high Positive Affective Engagement is more accurately detected in the German dataset, whereas English data better identifies low Perceived Mental Strain. This suggests that linguistic and cultural factors influence speech patterns associated with psychological states. The findings align with prior research that indicates variations in emotional expression and self-reporting tendencies across languages (29), affecting the reliability of cross-linguistic speech analysis.

The results underscore the importance of tailoring speech-based assessments to account for linguistic and cultural differences. The observed disparities suggest that speech processing models trained on one language may not generalize well to another, necessitating language-specific adaptations. This is particularly relevant for multilingual clinical applications, where accurate psychological state estimation is crucial. Future implementations should explore adaptive modeling approaches that incorporate linguistic variations into speech-based assessments.

Moreover, the study demonstrates that prosodic features such as pitch variability, loudness, and voice activity provide meaningful indicators of psychological states. These findings could inform the development of more robust emotion recognition systems, improving their reliability in real-world applications such as mental health monitoring and human-computer interaction.

Despite the promising findings, this study has several limitations. First, the dataset is relatively small, with only 130 participants across both language groups. A larger and more diverse sample could enhance the generalizability of the results. Additionally, cultural and contextual factors influencing Positive Affective Engagement and Perceived Mental Strain reporting were not explicitly controlled for, which could have influenced the differences. And, while the OpenSmile toolkit provides over thousands of features, incorporating all of them without prior selection would necessitate additional dimensionality reduction and introduce model complexity that may hinder interpretability. Future work may explore automated feature selection from the full feature set.

While the observed performance differences between English and German participants were attributed to prosodic variation, socio-cultural and attitudinal differences in self-reporting may also contribute to these effects. Previous studies have documented cross-cultural biases in self-assessment, with German participants often exhibiting more conservative or critical self-evaluations compared to English-speaking counterparts. Such biases could impact ground truth labels and therefore model outcomes.

Limitation also lies in the interpretability of the feature space. Although PCA reduced dimensionality effectively and preserved key prosodic patterns, it introduces a layer of abstraction that can obscure direct relationships with psychological constructs. While we report loadings and variance explained to improve transparency, future work should consider interpretable models such as LASSO or random forest regressors that maintain a clear mapping between original features and target variables.

Another limitation is the reliance on self-reported measures for Positive Affective Engagement and Perceived Mental Strain. Subjective assessments may introduce bias, as individuals perceive and report their psychological states differently. Incorporating objective physiological measures, such as heart rate variability or galvanic skin response, could provide a more comprehensive evaluation. Although SEWA ratings are subjective and lack psychometric standardization, our composite scores were informed by theoretical frameworks linking enjoyment and engagement to resilience, and cognitive effort to load. To support construct validity, future studies will incorporate validated scales such as the Brief Resilience Coping Scale (BRCS) and NASA-TLX alongside SEWA ratings for triangulation.

Furthermore, while this study focuses on English and German, the findings may not extend to other languages with distinct prosodic characteristics. Future research should explore additional languages to determine whether similar trends persist and refine cross-linguistic models accordingly. While SVMs and linear regression provide interpretable baselines, we acknowledge their limitations in capturing non-linear and sequential dependencies inherent in prosodic speech patterns. Preliminary work using Random Forests and LSTM-based architectures is underway to explore non-linear interactions and temporal modeling.

Building on the current findings, future research should address several key areas. Expanding the dataset with more participants and diverse demographic backgrounds would improve model robustness. Investigating alternative machine learning approaches, such as deep learning models, could enhance prediction accuracy by capturing complex non-linear relationships between speech prosody and psychological states (26). Moreover, Integration of global and local prosodic features has been shown to enhance the accuracy of emotion recognition systems. Global features capture overarching statistics like mean and standard deviation of prosodic contours, while local features represent temporal dynamics at finer granularities, such as syllables and words (52). The current study focuses on global prosodic features. However, to capture within-conversation variation in load and resilience, future work will incorporate temporal modeling of prosodic contours (e.g., pitch trajectories, pause timing) at the utterance level. Tools like Praat or Voice Activity Detection (VAD) will enable segmentation aligned with speech turns, allowing for dynamic load tracking across dialogue.

Additionally, exploring cross-linguistic transfer learning could help mitigate performance gaps between languages. Training models on a diverse set of languages and fine-tuning them for specific linguistic contexts could improve generalizability. Future studies should also consider incorporating multimodal data, such as facial expressions and physiological signals, to create a more holistic assessment framework. Finally, real-world validation of these models in clinical or everyday settings would provide practical insights into their effectiveness. Testing speech-based psychological assessment tools in naturalistic environments could help refine their application for mental health monitoring, cognitive workload analysis, and human-computer interaction.

It is important to note that, in this study, the spoken content was tied to an induced emotional state, as participants discussed advertising videos rather than reflecting on their mood in a general sense. This design enables greater experimental control over affective stimuli, but it may not fully capture the natural variability and authenticity present in spontaneous speech. In contrast, studies such as (53) focus on self-initiated, spontaneous speech to detect mental health risks such as depression and anxiety, offering richer insights into a person’s habitual emotional tone. Induced states may elicit different prosodic patterns compared to spontaneous self-disclosure, which should be considered when interpreting the findings. Nonetheless, this approach further highlights the potential of speech as a non-invasive biomarker for early detection of mental health conditions. From a clinical perspective, identifying reliable vocal indicators—even from neutral or task-oriented speech—could offer valuable insights into an individual’s psychological well-being without requiring explicit discussion of sensitive topics. This would be especially relevant in addressing the persistent stigma surrounding mental health issues, supporting unobtrusive monitoring and timely intervention.

In summary, this study demonstrates the impact of language on speech-based psychological assessments, with German data showing stronger predictability for Positive Affective Engagement (emotional resilience) while Perceived Mental Strain (cognitive load) prediction remains similar across languages. The results highlight the importance of language-specific speech features in machine learning models and underscore the need for tailored approaches in multilingual settings. Future research should address dataset limitations, explore alternative modeling techniques, and validate findings in real-world applications to enhance the effectiveness of speech-based psychological assessments.

## 5 Conclusion

This study explored the relationship between speech prosody and psychological constructs, specifically Positive Affective Engagement (emotional resilience) and Perceived Mental Strain (cognitive load), using English and German speech samples from the SEWA dataset. Through statistical analysis and machine learning models, we demonstrated that prosodic features — such as pitch variability, intensity, and spectral characteristics — serve as meaningful indicators of psychological states. Our results revealed notable language-based differences in speech characteristics and their correlation with emotional and cognitive attributes.

Machine learning models performed differently across the two languages, with linear regression yielding lower errors for Positive Affective Engagement (emotional resilience) in German, while Perceived Mental Strain (cognitive load) prediction remained relatively similar across datasets. Classification results from SVM indicated that high Positive Affective Engagement was more accurately detected in German, whereas low Perceived Mental Strain was better identified in English. These findings suggest that language-specific acoustic patterns influence the reliability of psychological inferences, emphasizing the need for linguistic adaptations in automated speech-based assessments.

While this study provides valuable insights, certain limitations must be acknowledged. The size of the data set was relatively small, and cultural differences in self-reporting may have influenced ground-truth labels. Future research should explore larger and more diverse datasets, apply deep learning techniques tailored to Positive Affective Engagement and Perceived Mental Strain detection, and investigate the generalization of findings across additional languages. By addressing these challenges, speech-based psychological assessment tools can be refined to enhance their accuracy and applicability in multilingual and cross-cultural contexts.

## Data Availability

Publicly available datasets were analyzed in this study. This data can be found here: http://db.sewaproject.eu/.
